# PlantTalk: A Smartphone-Based Intelligent Hydroponic Plant Box

**DOI:** 10.3390/s19081763

**Published:** 2019-04-12

**Authors:** Lan-Da Van, Yi-Bing Lin, Tsung-Han Wu, Yun-Wei Lin, Syuan-Ru Peng, Lin-Hang Kao, Chun-Hao Chang

**Affiliations:** Department of Computer Science, National Chiao Tung University, Hsinchu 300, Taiwan; liny@csie.nctu.edu.tw (Y.-B.L.); stevensuperboy.cs02g@g2.nctu.edu.tw (T.-H.W.); jyneda@nctu.edu.tw (Y.-W.L.); dz92286.me04@g2.nctu.edu.tw (S.-R.P.); sunrise510523.cs04@g2.nctu.edu.tw (L.-H.K.); neilchang.cs05g@g2.nctu.edu.tw (C.-H.C.)

**Keywords:** hydroponic planting, Internet of Things, plant actuator, plant factory, plant sensor, smartphone control

## Abstract

This paper proposes an IoT-based intelligent hydroponic plant factory solution called PlantTalk. The novelty of our approach is that the PlantTalk intelligence can be built through an arbitrary smartphone. We show that PlantTalk can flexibly configure the connections of various plant sensors and actuators through a smartphone. One can also conveniently write Python programs for plant-care intelligence through the smart phone. The developed plant-care intelligence includes automatic LED lighting, water spray, water pump and so on. As an example, we show that the PlantTalk intelligence effectively lowers the CO_2_ concentration, and the reduction speed is 53% faster than a traditional plant system. PlantTalk has been extended for a plant factory called AgriTalk.

## 1. Introduction

For urban dwellers living in a busy smog-filled city, an excellent way to relax is to grow houseplants. Indoor plants provide a lavish look and can be used to decorate the interior, which changes the look and feel of the home with limited space. Houseplants also help purify the air by absorbing carbon dioxide, removing chemical vapors, and offering transpiration rates to potentially improve the homeowner’s quality of life. Caring for houseplants may not be easy for a garden beginner or a busy homeowner. Due to the advance of Internet of Things (IoT) technology, plant sensors have become a great tool to provide valuable assistance. Consequently, many modern plant-care systems use sensors and actuators to measure and control plant growth to help gardeners maintain a proper environment to stabilize the production and quality of plants [1]. Kameoka et al. [2] used customized wireless temperature-humidity sensors in micro-miniature size to help a greenhouse keeper enhance the environment control. Ijaz et al. [3] achieved low power consumption by using the ZigBee protocol for sensor communications and replacing fluorescent lighting with LED lighting in a plant factory. Sugano et al. [4] developed a plant factory equipped with automatic cultivation system including robotic screening of seedlings on a cultivation panel, direct ventilation control, lighting systems with hybrid light source and a data analysis system. AeroGarden [5] emphasized a small-scale plant box, which possesses an LED system and a dashboard reminding the user to add water and nutrients into the plant tank. Kobayashi et al. [6] studied impact of various LED light sources on the growth of hydroponic lettuce. To save energy, Lee et al. [7] developed a system to switch light sources between LED and sunlight based on the intensity of sunlight radiation. To investigate the plant growth performance, Harun et al. [8] conducted experiments with a mixed light source by blue and red at ratio of 4:1.

Since smartphones are widely used, smartphone-controlled plant-care systems are emerging and attracting the research and the market. Siregar et al. [9] only allowed the gardener to monitor the sensor data through smartphone, and the user had to manually control the actuators. Pitakphongmetha et al. [10] and Ruengittinun et al. [11] implemented control applications that need to be installed in a smartphone. Therefore, not every smartphone can access the applications. Changmai et al. [12] implemented web-based monitoring applications but did not provide soft switches/buttons for users.

From the above literature review [1,2,3,4,5,6,7,8,9,10,11,12], it is observed that indoor smart planting has been very popular in the past four years. However, by the end of 2018, many smart plant sensor products had disappeared because consumers today want to know more about their plants than what this class of sensors can provide. To address this issue, we propose the PlantTalk approach that has the potential to create niche products and make plant sensors popular again. Our solution aims to achieve the following features.

**Feature 1**. All PlantTalk operations can be performed through an arbitrary smartphone or a computer. Features 2–4 below are provided by PlantTalk graphical user interface (GUI) through the browser of any smartphone. PlantTalk GUI is designed specifically for smartphone operations. This powerful feature allows the designers and the users to access PlantTalk at any time in any place.
**Feature 2**. PlantTalk provides options so that the user can flexibly select the off-the-shelf IoT devices to be included or changed in their hydroponic plant factories. In particular, an off-the-shelf camera can be integrated into PlantTalk, which allows the user to remotely view the plants when they are manipulated with the IoT devices in real time.
**Feature 3**. PlantTalk allows sharing of IoT devices among applications. For example, for a homeowner who has both a hydroponic plant box and a smart aquarium tank, PlantTalk allows sharing of the reverse osmosis (RO) for both applications (i.e., the RO can be used and controlled by both applications at the same time) if the water is connected in the plant box and the aquarium tank.
**Feature 4**. PlantTalk intelligence can be easily created and maintained, which allows the users to know more about their plants than what the sensors can provide. That is, the sensed data are not only shown to the users, but also used to drive the actuators for meaningful actions.

PlantTalk is an IoT-based intelligent hydroponic plant factory that grows plants without actually having to plant them in the ground. Our approach grows plants through a nutrient-rich water solution without the use of traditional dirt medium. PlantTalk has been extended to a large-scale plant factory called AgriTalk [13]. This paper uses a plant-care box and Devil’s Ivy as an example to show how PlantTalk works. Devil’s Ivy is rated one of the best houseplants for removing all indoor air toxins (such as benzene, trichloroethylene, xylene, and formaldehyde) [14], and is therefore considered in this paper to demonstrate how the plant purifies the air.

Among the existing smart plant approaches [2,3,4,5,6,7,8], PlantTalk is the only solution that provides smartphone-based intelligence to support Features 1–4. That is, the PlantTalk mechanism is carefully tailored with web and user experience design such that its intelligence can be easily operated through a browser-equipped mobile device. Table 1 summarizes the functions of the smart plant-care systems [2,3,4,5] described above. Except for PlantTalk, the solutions in Table 1 do not elaborate on how to easily and quickly add/remove/exchange sensors and actuators. Unlike PlantTalk, none of the smartphone-controlled plant-care systems [9,10,11,12] allow integration of a camera in their smartphone applications to remotely observe the plants taken care of by the sensors and the actuators. These solutions [9,10,11,12] partially implemented Feature 2 (only supporting Arduino control board). They also partially implemented Feature 4 to allow the gardener to view the sensor readings and control the actuators through smartphones. The designer cannot implement control intelligence through smartphones. Furthermore, the plant boxes in these solutions are prototypes for a demo. On the other hand, the PlantTalk plant box and its IoT system are implemented for service trials and can be considered as commercial plant factory products. We will elaborate on PlantTalk’s unique IoT intelligence for plant care in the subsequent sections.

The paper is organized as follows. Section 2 describes the PlantTalk intelligent hydroponic plant-care system. Section 3 elaborates on the plant sensors and actuators used in PlantTalk. Section 4 shows how a PlantTalk project can be efficiently built by using the browser of a smartphone. Section 5 investigates how PlantTalk purifies the air. The discussion and conclusion is made in Section 6.

## 2. The PlantTalk Intelligent Hydroponic Plant-Care System

**Figure 1:**
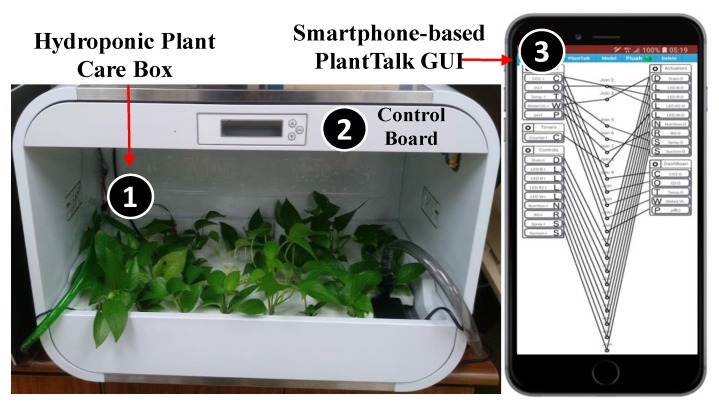
The hydroponic plant box and the PlantTalk GUI.

**Figure 2:**
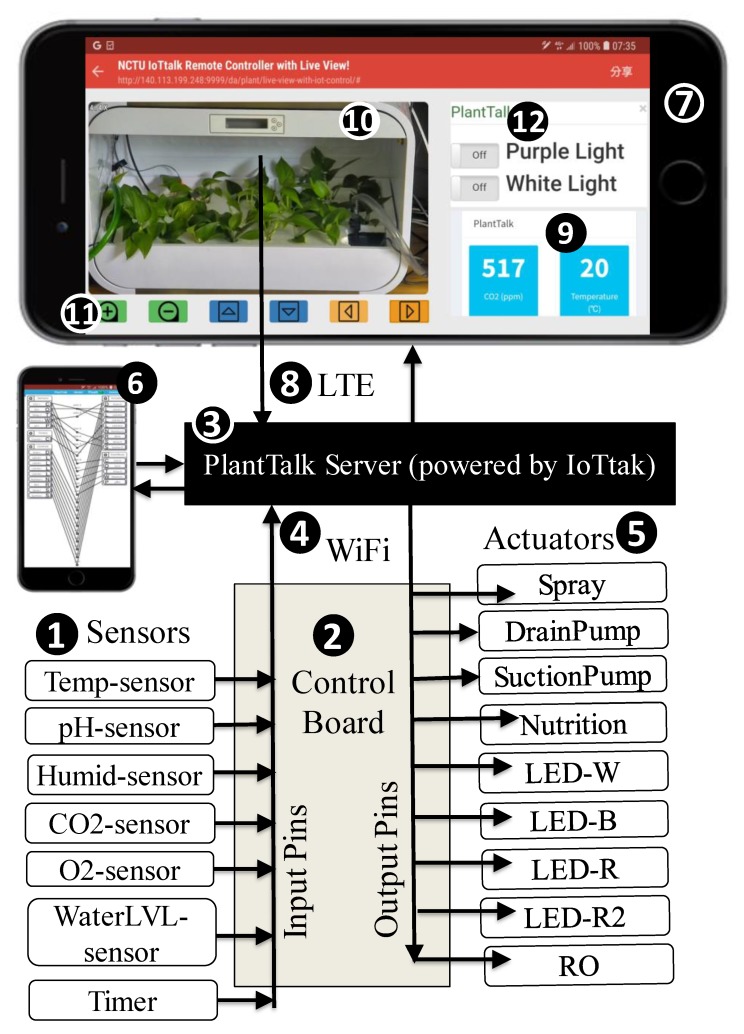
PlantTalk functional block diagram.

Built on top of the IoT management platform called IoTtalk [15], we develop the PlantTalk system that integrates the hydroponic plant box (Figure 1 (1)) through a control board (Figure 1 (2)). The connections of all plant IoT devices can be set up through the web-based PlantTalk GUI by using the browser of any smartphone (Figure 1 (3)). Figure 2 illustrates the functional block diagram for PlantTalk. In this figure, the plant sensors (Figure 2 (1)) send the measured data to the control board (Figure 2 (2)) through the input pins of the control board. The control board sends the data to the PlantTalk server (Figure 2 (3)) through Ethernet or Wi-Fi (Figure 2 (4)). The PlantTalk server processes the received data and then gives instructions to drive the plant actuators (Figure 2 (5)) through the output pins of the control board. Please note that if the number of IoT devices connected to the control board is larger than the pin number in the board, then multiple control boards are used. Based on the work in [13], PlantTalk can transparently support multiple control boards without extra effort. In the current implementation, the PlantTalk server is in both the cloud of ChungHwa Telecom and the university campus center for fault tolerance purposes. The intelligence of the server can be built through the browser of a smartphone (Figure 2 (6); see also Figure 1 (3)) or any computer.

**Figure 3:**
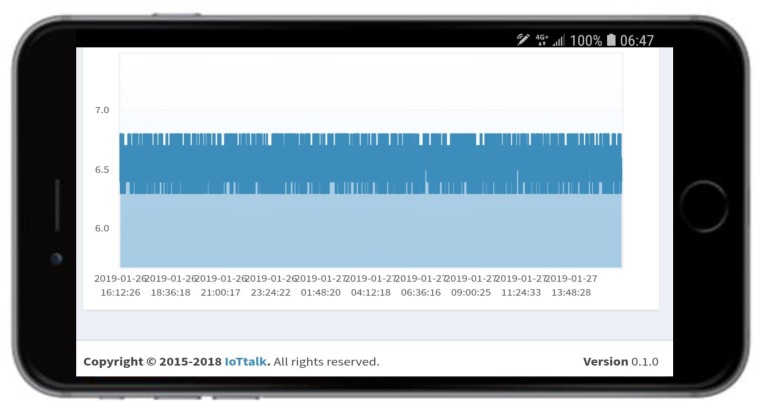
The time series chart for the pH level.

The PlantTalk server can also send sensor data to any smartphone (Figure 2 (7)) through the LTE or 5G wireless technologies (Figure 2 (8)). Then the user can view the data through the phone’s browser (Figure 2 (9)). Optionally, PlantTalk can accommodate a camera so that the user can enjoy viewing the beautiful plants or see the results of controlling the plant actuators (Figure 2 (10)) remotely through the smartphone. The video control bar (Figure 2 (11)) allows the user to move, rotate and zoom the camera. The smartphone also provides soft switches (Figure 2 (12)) that allows the user to control the actuators in the hydroponic plant box. For example, if the user clicks the red LED button, the smartphone will turn on the red LED through the path ((12)-> (3)-> (2)-> (5)). The smartphone for the server (Figure 2 (6)) and the smartphone for control and display (Figure 2 (7)) can be the same one. For the measured data of each sensor, PlantTalk logs the history, and the user can see the time series chart of a sensor by clicking this sensor’s icon in the dashboard (Figure 2 (9)). When the icon is clicked, the time series chart is shown in the smartphone. For example, Figure 3 shows the time series chart of the pH level.

The major hardware of PlantTalk is the control board (Figure 2 (2)). PlantTalk supports various control boards including Arduino, ESP8266 ESP-12F, ROHM IoT kit, and MediaTek LinkIt Smart 7688 duo. Any sensors and actuators connected to these control boards can be directly accommodated by PlantTalk. PlantTalk provides device application software that can be installed in the above control boards for communication with the PlantTalk server. It is our goal to make PlantTalk an open platform to accommodate as many plant sensors and actuators as possible. Our solution supports off-the-shelf IoT products through various control boards.

We note that a PlantTalk user is a gardener or a homeowner who enjoys plant care through the soft switches/buttons and the camera of an arbitrary smartphone (Figure 2 (7)). On the other hand, the PlantTalk designer can be an ICT (Information and Communication Technology) engineer who creates and maintains projects through the PlantTalk GUI (Figure 2 (6)).

## 3. Plant Sensors and Actuators

This section describes the plant sensors, the controls and the plant actuators used in PlantTalk.

### 3.1. Plant Sensors

The current version of PlantTalk includes the plant sensors for pH, temperature, humidity, water level, CO_2_, and O_2_. There is also a software timer and several soft control switches/buttons.

The pH sensor (pH-sensor in Figure 2 (1)) indicates the quality of the water. The optimum pH levels of the water for different plants are not the same. Water with wrong pH level can significantly reduce the ability of the plants to absorb carbohydrates and vitamins, as well as micronutrients and other beneficial sources. For root growth and regeneration, alkaline water or hard water is not suitable for mist. The optimum pH of the water for misting is 5.5 to 6.5. In Figure 3, the time series chart indicates that the pH level is too high and must be reduced.

The temperature and the humidity sensors (Humid-sensor and Temp-sensor in Figure 2 (1)) are used to sense the temperature and the humidity of the plant box. Like most plants, Devil’s Ivy prefers a temperature between 60–80 °F. It will withstand occasional low temperature down to 50 °F, but the plant will die below this temperature. Devil’s Ivy grows better with a little bit more humidity. Brown leaf tips may indicate that the air in the room is too dry. Misting the plant leaves will prevent the dryness of leaves by increasing humidity.

Water loses oxygen over time, and we should pour out the old water and add in fresh water every month. Sometimes it is good that roots or sections of roots are exposed to the air. However, most of the roots should be submerged below water. The water level sensor (WaterLVL-sensor in Figure 2 (1)) indicates when and how much water should be added. The CO_2_ and the O_2_ sensors (CO_2_-sensor and O_2_-sensor in Figure 2 (1)) are used to see how good Devil’s Ivy can refresh the air. More details will be given in Section 5.

In PlantTalk, the actuators are automatically controlled by the sensors described in this subsection. Furthermore, we implement a soft switch/button (Figure 2 (12)) per actuator supported by PlantTalk for manual control. We also implement a software timer (Timer in Figure 2 (1)) that can trigger the actuators for routine jobs.

### 3.2. Plant Actuators

The current version of PlantTalk actuators includes sprayer, drain pump, suction pump, nutrition pump, and several LEDs. There is also a dashboard to display all sensor data.

Plants grown in water still need sunlight, ideally at least six hours a day. Indoor hydroponic systems are often located in places without access to direct sunlight, and therefore needs to equip with the necessary light fixtures. In our example, Devil’s Ivy cannot bear full, direct sun and complete darkness. On the other hand, it prospers well in both bright and dim lighting inside the house. When Devil’s Ivy exposes to bright, filtered light, it will have more yellow variegation in its leaves. In PlantTalk, LED lighting actuators (LED-W, LED-B, LED-R, and LED-R2 in Figure 2 (5)) are used to provide appropriate lighting.

One of the most versatile mechanisms for hydroponics nutrition is the Nutrient Film Technique (NFT) that is operated by the nutrition and water pumping actuators (Nutrition, DrainPump and SuctionPump in Figure 2 (5)). PlantTalk uses a suction motor to pump the water containing dissolved nutrients into a grow tray, so that the nutrients can be absorbed by the plants as the water passes through their roots. A drain motor then pumps the water to a lower reservoir, which is eventually pumped back to the grow tray. NFT is particularly useful for lightweight and fast-growing plants such as lettuce.

Hydroponic fertilizers should be used according to package directions that are different for flowering plants, leafy vegetables or others. The nutrients can be organic or synthetic, which include both macronutrients (nitrogen, potassium, phosphorus, calcium, and magnesium) and micronutrients (trace amounts of iron, manganese, boron, zinc, copper, molybdenum, and chlorine). The nutrients are typically diluted to about one fourth of the recommended strength on the plant box, and the diluted mixture is added into the plant box every four to six weeks. Please note that the garden fertilizers are formulated for use in garden soil, which should not be used in a hydroponic system.

RO actuator (RO in Figure 2 (5)) is an economical water treatment system. Water obtained from an RO system is almost as good as expensive distilled water for the nutrient solution. The RO water changes the tap water quality to be in the “sweet spot” pH range, i.e., between 5.5 and 6.3.

When the air in the room is too dry, the sprayer (Spray in Figure 2 (5)) should be activated to mist the plant leaves to improve humidity and prevent the dryness of leaves. The sprayer is also used to lower the temperature.

PlantTalk provides a special display actuator called the dashboard (Figure 2 (9)). The dashboard allows a user to see the up-to-date value of a sensor as well as its time series chart (e.g., Figure 3) by clicking this sensor’s icon in the dashboard.

## 4. Configuring PlantTalk Projects

**Figure 4:**
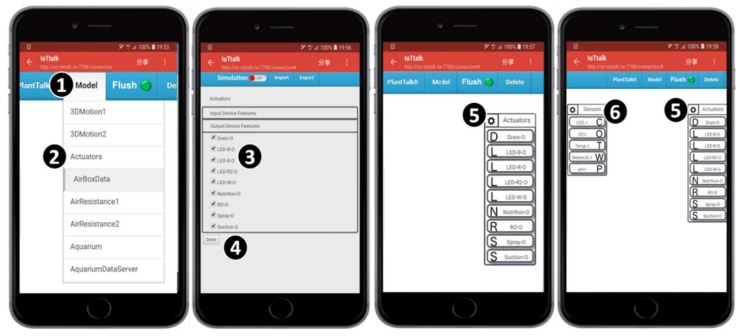
Configuring a PlantTalk project.

An arbitrary smartphone can access the GUI of the PlantTalk server through its browser (Figure 1 (3)). In the web-based GUI, the “Model” pulldown menu bar (Figure 4 (1)) contains all IoT devices that can be accommodated in PlantTalk. When the Actuators item is selected (Figure 4 (2)), a window pops up to show all plant actuators that can be used. The window provides check boxes to select the actuators (see Figure 4 (3)).

**Figure 5:**
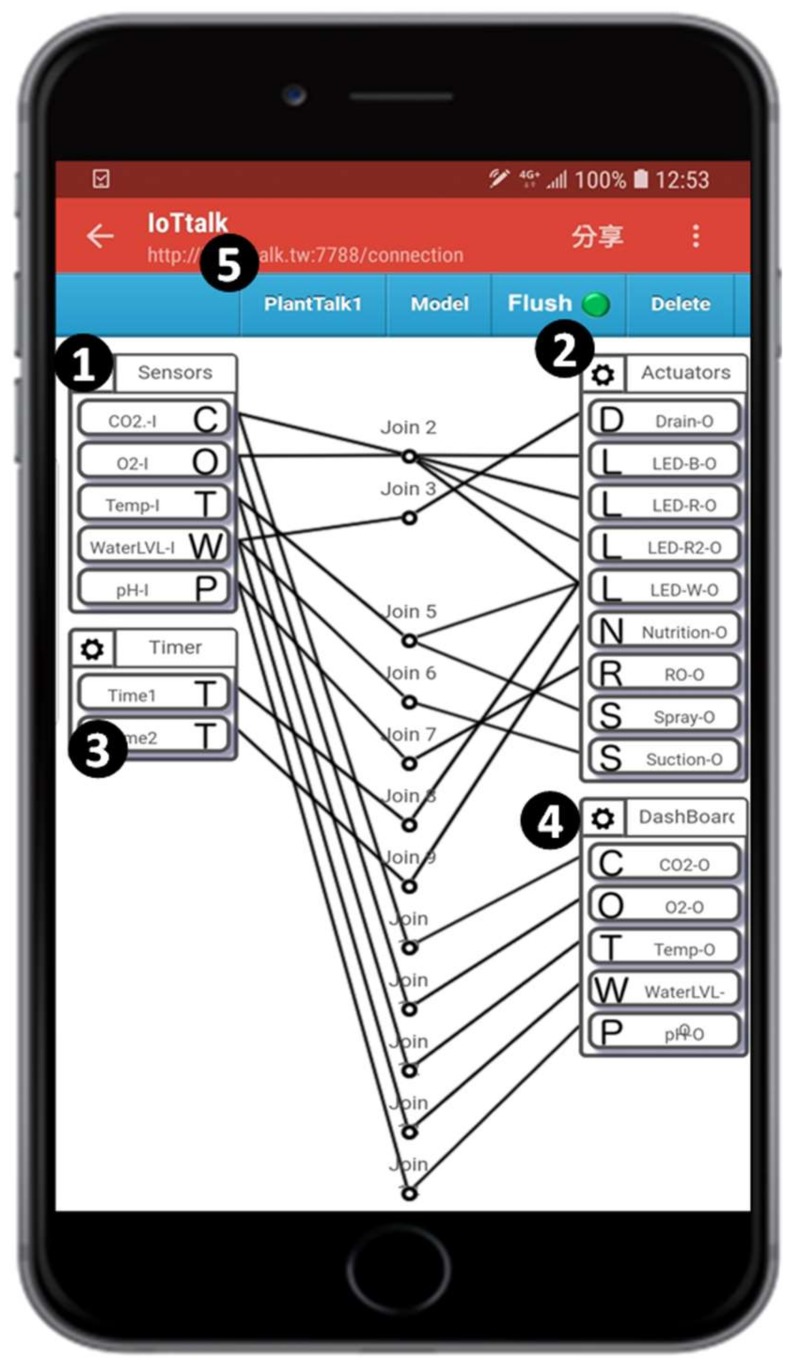
Project PlantTalk1.

In this example, 9 actuators are selected. After the actuators have been selected and the “Save” button is pressed (Figure 4 (4)), the icon for Actuators is shown in the GUI window (Figure 4 (5)). Inside the Actuators icon, there are 9 icons representing the selected actuators named Spray-O, Drain-O, Suction-O, Nutrition-O, LED-W-O, LED-B-O, LED-R-O, LED-R2-O, and RO-O. Similarly, we create the Sensors icon with 5 sensors (Figure 4 (6)): Temp-I, pH-I, CO_2_-I, O_2_-I, and WaterLVL-I. From the viewpoint of PlantTalk, “Sensors” is an input IoT device that sends data to the PlantTalk server, and “Actuators” is an output IoT device that receives instructions from the server. The sensors inside Sensors are called input device features (IDFs), and their names in the icons are appended with “-I”. Similarly, the actuators inside Actuators are called output device features (ODFs), and their names in the icons are appended with “-O”. In PlantTalk, an input device is placed in the left-hand side of the GUI window, and an output device is placed in the right-hand side of the window. We continue to create the Timers input device (Figure 5 (3)) and the Dashboard output device (Figure 5 (4)).

To interact an IDF with an ODF, we simply drag a line between the IDF icon to the ODF icon. For example, the link “Join 5” allows the temperature sensor Temp-I to control the sprayer through the Spray-O ODF. A Join connection consists of two segments connected to a circle. The circle represents a Python function that provides intelligence to handle the data sent from the input device to the PlantTalk server through the left-side line segment, and the intelligence to create the instruction of the server given to the output device through the right-side line segment. After all connections have been built, we save this configuration with the name PlantTalk1 (Figure 5 (5)).

Project PlantTalk1 includes several automatic control mechanisms. The water control is achieved by Joins 3 and 6 in Figure 5. The water level sensor WaterLVL-I detects the water volume in the sink. Whenever the water level is lower than the low-level threshold, through Join 6, the suction pump starts to draw water into the plant box until the water level is higher than the threshold. Similarly, when the water level is higher than the high-level threshold, through Join 3, the drain motor starts to pump the water out of the plant box until the water level is lower than the threshold.

When the temperature sensor Temp-I detects that the temperature is higher than a threshold, the sprayer (Spray-O) is activated through Join 5 to spray water on plant leaves to lower the temperature. Also, Join 5 turns off the white light (LED-W-O) to reduce the light heat.

The white light is periodically turned on by the timer Time1 through Join 8 for routine operation. The white light is turned off when the purple light is turned on. This control is achieved through Join 2, and the details are given in the next section.

Through Join 7, the RO-O is triggered by pH-I to transform the tap water in the pH range between 5.5 and 6.3. In PlantTalk, nutrient supply follows a user-defined time period to trigger a pump that draws nutrient into the sink in PlantTalk. The time to supply nutrient is set by the countdown timer Time2, which triggers a pump that draws nutrient into the sink in the plant box through Join 9.

In each of these connections, the intelligence is provided by the corresponding Join function. We will show how Join functions are created in the next section. In the PlantTalk1 project, all sensor data are sent to the Dashboard output device for display (the browser of a smartphone) through five Join connections between Figure 5 (1) and Figure 5 (4).

**Figure 6:**
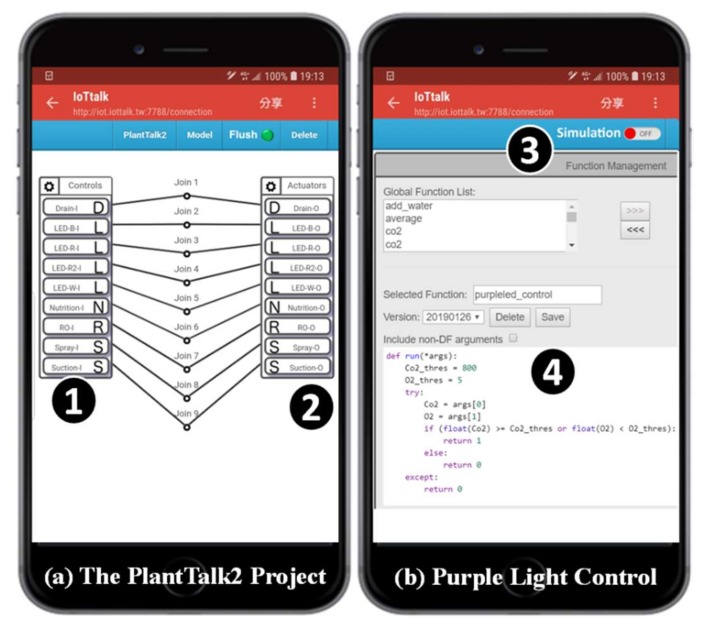
PlantTalk project 2 and the function management window.

To manually control the plant actuators through an arbitrary smartphone, we build a second project PlantTalk2 (Figure 6 (a)) with the input device “Controls” (Figure 6 (1)) connected to the Actuators output device (Figure 6 (2)). The Actuators icons in Figure 5 (2) and Figure 6 (2) represent the same set of actuators. In this way, these actuators can be controlled by both projects PlantTalk1 and PlantTalk2 at the same time.

The message delivery/processing delays in PlantTalk are short and real-time responses can be achieved. For a NB-IoT communication system, the expected delay E[$t_{N,s}$] to send a message from a sensor to the PlantTalk server is E[$t_{N,s}$] = 0.3792 s and the variance V[$t_{N,s}$] = 0.5013 E[$t_{N,s}$]^2^, and the expected delay E[$t_{N,a}$] for sending a message from the PlantTalk server to an actuator is E[$t_{N,a}$] = 1.6461 s and V[$t_{N,a}$] = 0.1363 E[$t_{N,a}$]^2^. For Wi-Fi access, the expected delay E[$t_{w}$] for sending an message from a sensor to the PlantTalk server and sending a message from the PlantTalk server to an actuator are the same, where from 1000 measurements, E[$t_{w}$] = 31.68 ms and V[$t_{w}$] = 0.0022 E[$t_{w}$]^2^ [16].

## 5. Air Cleaning Experiments

This section shows a smartphone-based intelligent application for air cleaning. With appropriate setup for sensors and actuators, PlantTalk enhances the air quality through LED lighting. In presence of light, the photosynthesis process uses CO_2_ and water to produce carbohydrates and O_2_. Different colors of light have different effects on plants except for green light. Plants do not absorb green light, and plant leaves appear green by reflecting green light. With blue light (LED-B-O in Figure 5 (2)), plants germinate seeds and seedlings, and grows leaves, stalks, and stems. Red light (LED-R-O) is needed for the production of fruit and flowering. Also, plants get more energy from purple light than the same amount of light from other colors.

**Figure 7:**
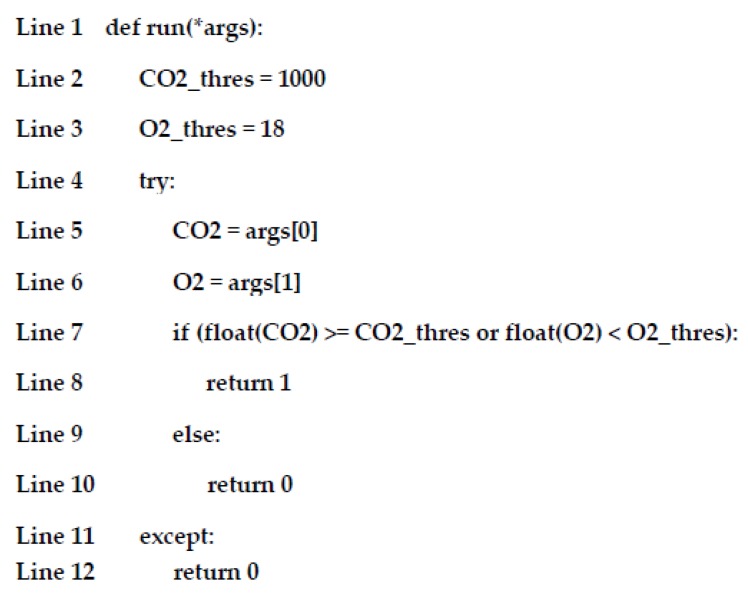
Function code in the experiment.

To purify the air in PlantTalk (i.e., to reduce the CO_2_ concentration and increase the O_2_ concentration), the purple light is controlled by both the O_2_ and the CO_2_ sensors through Join 2 in Figure 5. With this connection, PlantTalk gives instruction to mix two red LED (LED-R-O and LED-R2-O) and one blue LED (LED-B-O) to produce purple light. Air purification is activated if the CO_2_ concentration is higher than or equal to 1000 ppm or the O_2_ concentration is lower than 18%. To implement the air purification function, we click the Join 2 circle in Figure 5, and a function management window pops up (Figure 6 (3)), where one can write a Python program (Figure 6 (4)) in the smartphone or any computer. The function code used in this experiment is listed in Figure 7:

In Line 1, *args are the inputs, where args[0] receives the data sent from CO2-I and args[1] receives the data sent from O_2_-I. Lines 2 and 3 set the CO_2_ threshold (1000 ppm) and the O_2_ threshold (18%). Line 7 checks if the conditions for turning on the purple light are met. If so, Line 8 returns 1 (turn-on). Otherwise, Line 10 returns 0 (turn-off).

To see how the air purification function works, we conduct the following experiments on Devil’s Ivy [1]. There are two scenarios. Scenario 1 is the baseline where PlantTalk’s LED lighting control is disabled. Scenario 2 supports PlantTalk intelligence where lighting control is activated.

**Figure 8:**
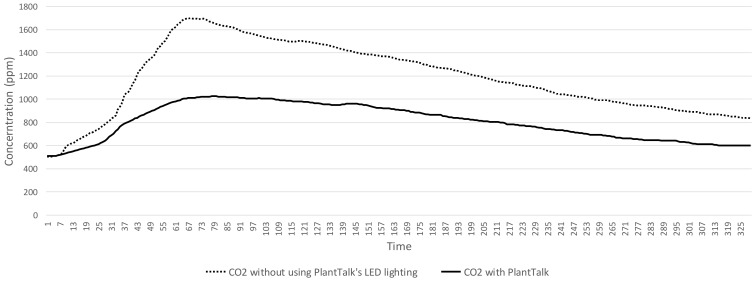
CO_2_ reduction experiment.

**Figure 9:**
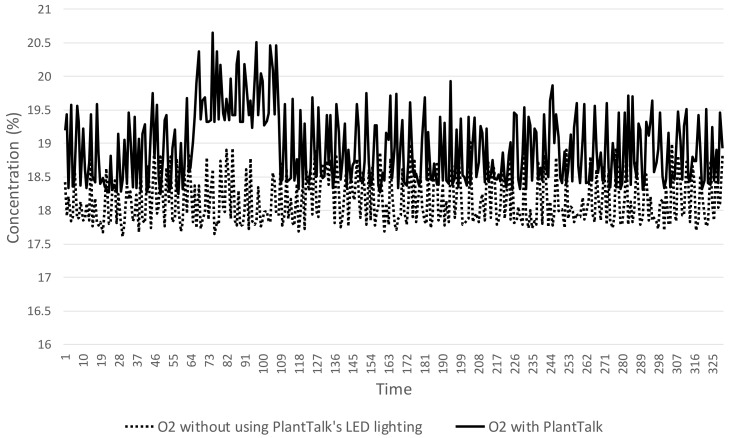
O_2_ improvement experiment.

In our experiments, 0.6 gm ± 0.1 gm of baking soda and vinegar are used to produce the same CO_2_ amounts for both scenarios. In Scenario 1, the maximum CO_2_ concentration reaches 1699 ppm in Figure 8. In Scenario 2, the maximum CO_2_ concentration reaches 1026 ppm in Figure 8. The maximum CO_2_ reduction in Scenario 2 is less than that in Scenario 1 by 673 ppm. The speed of CO_2_ concentration reduction down to 1000 ppm in Scenario 2 is 53% faster than that for Scenario 1. The second experiment measures the distribution of the O_2_ concentration in Figure 9, which indicates that O_2_ concentration for Scenario 2 ranges from 18% to 21%, which is higher than that for Scenario 1 most of the time. Specifically, the O_2_ concentration in Scenario 2 is always higher than that in Scenario 1.

PlantTalk provides control functions beyond “thresholds”. For example, we can predict the germination probability of rice blast spore to automatically treat pesticide for preventing disease. The prediction probability is computed in a “non-threshold” Join function.

We are implementing another smartphone-based intelligent application by using PlantTalk. We will show how to use smartphone to quickly implement spore germination function and configure it as a PlantTalk feature. The spore germination function is used to detect rice blast, and the details will be given in a separate paper.

## 6. Discussion and Conclusions

This paper presented PlantTalk, an intelligent hydroponic plant-care system. We showed that PlantTalk has the potential to create niche indoor planting products with four features. Among them, Feature 1 is unique and has not been found in the existing solutions, where PlantTalk is designed such that Features 2–4 can be easily accessed through the browser of a smartphone. Therefore, the PlantTalk designer and the user can access PlantTalk at any time in any place.

In Feature 2, PlantTalk provides options so that the users can flexibly select appropriate IoT devices to be included or changed in their hydroponic plant boxes. This feature can be achieved because PlantTalk is an open platform that provides an application programming interface (API) to accommodate various types of control boards connected to off-the-shelf sensors and actuators manufactured by various vendors.

**Figure 10:**
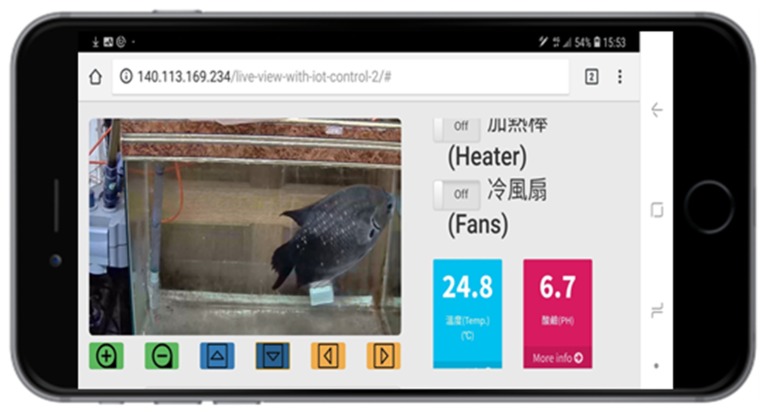
The video, dashboard and controls for the aquarium applications.

In Feature 3, PlantTalk allows sharing of IoT devices among applications. For example, if a homeowner has both a hydroponic plant box and a smart aquarium tank, PlantTalk allows sharing of the water pumps and the RO for both applications if the water is connected in the plant box and the aquarium tank. This feature is provided by the “Model” pulldown menu in the PlantTalk GUI. Another expensive equipment typically shared by different IoTtalk applications is the camera. Suppose that we install one camera in the room with PlantTalk and, e.g., smart aquarium tank. When the homeowner uses the smartphone to move the camera to the plant box, the PlantTalk video, dashboard and controls are shown (Figure 2 (7)). When the camera is moved to the aquarium tank, then the aquarium dashboard [16] and the controls are shown in the web window (see Figure 10).

With Feature 4, PlantTalk does not simply show raw data provided by the sensors. Its intelligence can be easily created to manipulate the raw data for driving the actuators to show interesting results so that the users know more about their plants. We used air cleaning as example to demonstrate this feature, and showed that the speed of CO_2_ concentration reduction for PlantTalk is 53% faster than that for a baseline plant-care scenario. We also showed that with the PlantTalk intelligence, O_2_ concentration can be improved up to 21%, which is higher than that of the baseline scenario. The homeowner can see the good results produced by the plants in the box through the time series charts shown in the smartphone.

Features 2–4 can be easily created and maintained by a PlantTalk designer, and be enjoyed by the users (customers and homeowners). Power of smartphone-based intelligence provided by Features 1 and 4 can be further demonstrated in the error control example. When many plant sensors and actuators are installed in a plant box (or plant factory), it is very often that some connections are not correct or inappropriate, and wrong or undesirable interaction between sensors and actuators may cause serious problems (e.g., water flooding). With Feature 1, we can remotely and visually monitor the result of wrong connections through a smartphone, and then use Feature 4 to modify the connections/functions through the same smartphone immediately (and the mistakes can be corrected immediately). The above example indicates that PlantTalk failure management can be operated by a standard smartphone. Here we emphasize that smartphone plays an important role to deal with real-time failures. As long as we have a smartphone, we can deal ALL PlantTalk errors (include programming) at any time in any place. Such smartphone-based intelligence has not been found in any IoT applications (including smart plant care).

Based on PlantTalk, we have developed a plant factory solution called AgriTalk [13] that was successfully recognized by potential customers and venture capitalists (VCs) at the CES (Consumer Electronics Show) held on 8–11 Jan 2019 in Las Vegas, Nevada, USA. Based on the VCs, the forecast initial market value for AgriTalk is about 30 million USD that agri-investors from around 10 countries are willing to collaborate with us to deploy AgriTalk in their countries. Smartphone-based intelligence is the key to control large-scale plant areas. With PlantTalk, a farmer can monitor and control farm activities in multiple areas at any time in any place. Right now, we have exercised PlantTalk in soil cultivation of size 600m^2^, where the sensors are installed in a micro-weather climate.

## Figures and Tables

**Table 1:** Comparisons of Plant-Care Systems.

System	Kameoka	Ijaz	Sugano	AeroGarden	PlantTalk
Factory/Farm (F) or Home (H)	F	F	F	H	H
Hydroponic (H) or Soil (S)	S	H	H	H	H
IoT Device Management System	No	No	No	No	Yes
Smartphone-based Intelligence	No	No	No	No	Yes
CO_2_ Sensor	Yes	Yes	Yes	No	Yes
O_2_ Sensor	No	No	No	No	Yes
Lighting	Yes	Yes	Yes	Yes	Yes
Temperature	Yes	Yes	Yes	No	Yes
Humidity	Yes	Yes	Yes	No	Yes
pH	No	No	Yes	No	Yes
RO	No	No	No	No	Yes
Off-the-shelf IoT devices	No	Yes	No	No	Yes
